# Assessing diabetes mellitus knowledge among Syrian medical students: A cross-sectional study

**DOI:** 10.1016/j.heliyon.2021.e08079

**Published:** 2021-09-27

**Authors:** Fatema Mohsen, Homam Safieh, Mosa Shibani, Hlma Ismail, Mhd Amin Alzabibi, Humam Armashi, Bisher Sawaf

**Affiliations:** a Faculty of Medicine, Syrian Private University, Damascus, Syria; b Internal Medicine Department, Hamad General Hospital, Hamad Medical Corporation, Doha, Qatar; c Department of Internal Medicine, Faculty of Medicine, Syrian Private University, Damascus, Syria

**Keywords:** Awareness, Knowledge, Medical students, Syria, Diabetes mellitus

## Abstract

**Background:**

Diabetes mellitus is the fastest growing global health emergency of the 21st century. The Middle East and North Africa region have the highest prevalence of diabetes in the world. Since medical students are the pillars of future healthcare systems, their knowledge of the disease must be evaluated, updated, and enhanced appropriately.

**Methods:**

A cross-sectional study was conducted at the Syrian Private University (SPU) in November 2019 on World Diabetes Day in Damascus, during the Syrian war crisis. Data were collected through self-administered questionnaires and analyzed using the Statistical Package for Social Sciences version 25.0 (SPSS Inc., United States).

**Results:**

Of the 275 students, 74 (26.9%) were preclinical students and 201 (73%) were clinical students with a mean age of 21.9 (±3.70) years. 67 (25.0%) are overweight, and 26 (9.7%) are obese. Students revealed a good level of knowledge regarding clinical features, risk factors, and complications; however, a lack of knowledge was noticed in the general information and the diagnostic criteria section. Clinical year students (4th, 5th, 6th) demonstrated higher levels of awareness compared to students in pre-clinical years (1st, 2nd, 3rd).

**Conclusions:**

Knowledge and awareness of medical students about diabetes mellitus were found to have some gaps. Health education efforts are required to reinforce its identification and management at all levels, while also encouraging lifestyle modifications among our students.

## 1 Introduction

Diabetes mellitus (DM) is a group of metabolic disorders that are characterized by persistent hyperglycemia, and categorized into 3 main subtypes: type 1 DM, type 2 DM (which constitutes 90% of cases), and gestational DM [1]. DM is the fastest growing global health emergency of the 21st century [1]. 2019 estimates of DM prevalence among adults was 463 (9.3%) million, and is predicted to rise to 578 (10.2%) million by 2030 [1]. Of the 463 million people living with DM, 50.1% are unaware of their chronic manifestation, and 84.3% of the undiagnosed cases were found in low and middle-income countries [1]. The Middle East and North Africa region (MENA) has the highest prevalence of diabetes in the world; 12.2% of the population is estimated to be diabetic, and this figure is projected to rise to 13.3% by 2030 (world-age standardized) [1].

Approximately 4.2 million adults aged between 20–79 years, died as a result of DM and its complications in 2019, equivalent to 1 death every 8 s [1]. The World Health Organization (WHO) and the United Nations (UN) have set global targets to improve care, reduce premature death from diabetes, achieve universal health coverage, and provide universal access to affordable essential medications by 2030 [2]. These are important steps towards guaranteeing access to affordable high-quality care and alleviating financial catastrophe for the 578 million who will then be living with diabetes [1].

The WHO's Syrian Arab Republic profile for 2016 reported the prevalence of DM as 11.9%; as for the prevalence of DM-associated risk factors, 21.6% of the population were obese and 55% were overweight [3]. These rates are considerably higher than the worldwide average of 13% and 39% for obesity and overweight, respectively [4]. These risk factors, along with sedentary lifestyles, positive family history, smoking cessation, and environmental factors are triggers for DM [5].

As of 2016, there were no operational policies in place on a national level to monitor and prevent DM, reduce overweight and obesity, and reduce physical inactivity [3]. The Syrian crisis, now in its 10th year, has taken a considerable toll on the healthcare system. The war has damaged and destroyed hospitals and clinics throughout the country, caused a massive outflux of healthcare workers, and drastically reduced the production and availability of medications. 60% of insulin-dependent Syrians are at risk, as the only insulin-producing facility has been damaged and forced to halt production [6]. All of this has resulted in a healthcare system incapable of adequately stemming the tide of DM and its major risk factors. The lack of national screening and prevention strategies means that physicians and patient awareness must be our first line of defence against diabetes. Increasing the number of healthcare professionals who encourage community-based lifestyle modifications such as physical activity, a balanced diet, and screening high-risk groups can delay or prevent the onset of type 2 DM [7]. This training must start during medical school to alter future practices.

As there are no studies among Syrian medical students on the awareness of DM, this is the first study to be conducted during the Syrian war crisis. This paper aims to measure awareness and general knowledge of DM among Syrian Private University (SPU) medical students. The objectives of the study are to gauge specific knowledge around risk factors, clinical features, diagnosis, complications, prevention, and treatment as well as to ascertain whether the clinical curriculum adequately prepares medical students to confidently identify those at risk to delay the progression of DM.

## 2 Methods

### 2.1 Study design, setting, and participants

A cross-sectional study using convenience sampling was conducted by the faculty of medicine, Syrian Private University (SPU) in Damascus, Syria on World Diabetes Day (14/11/2019). A pilot study was conducted on 30 students to evaluate the clarity, reliability, relevance, and acceptability of the survey. This sample was excluded from the final sample to avoid bias. A paper-based questionnaire was administered to students in the faculty's assembly hall. Students were informed that their participation was voluntary, all of their responses were recorded anonymously, response to all questions was not mandatory, and were allowed to withdraw from participation at any time. We used a self-administered English-language questionnaire that was modelled after several published studies [8, 9]. The first section of the questionnaire contained 10 questions about socio-demographic information including gender, age, body mass index (BMI), social status, residence, university year, mother's level of education, smoking, alcohol consumption, and grade point average (GPA). The participants were then directed to complete the second part of the questionnaire consisting of 45 questions divided into 7 sections: general information (4 questions), risk factors (7 questions), signs and symptoms (7 questions), diagnosis (3 questions), complications (8 questions), prevention (6 questions), and treatment (2 questions). Ethical approval was obtained from the Institutional Review Board (IRB), Faculty of Medicine, Syrian Private University. The questionnaire is available in appendix 1.

### 2.2 Reliability analysis

Cronbach's alpha test was applied to determine the internal consistency of the questionnaire. The Cronbach's alpha value of the Arabic questionnaire was 0.664. The items were considered to represent an acceptable level of internal consistency [10].

### 2.3 Statistical analysis

Data were analyzed using the Statistical Package for Social Sciences version 25.0 (SPSS Inc., Chicago, IL, United States). Frequencies and percentages (for categorical variables) or means, medians, and standard deviations (SD) (for continuous variables) were reported. Chi-square test was performed to compare knowledge between preclinical and clinical students in 6 sections: general information, risk factors, clinical features, diagnosis, complications, treatment, and diet. p-*values* < 0.05 were considered statistically significant.

## 3 Results

### 3.1 Socio-demographic characteristics

Of 275 undergraduate medical students who completed the questionnaire, 74 (26.9%) were preclinical students and 201 (73%) were clinical students with a mean age of 21.9 (±3.70) years. Their ages ranged from 18 to 30 years with the majority being 20 to 25 (±5) years and living in the city 251(91.3%). 96(34.9%) smoke, 28 (10.2%) consume alcohol, 144 (52.4%) exercise regularly, 164 (61.2%) have a BMI within the reference range (between 18.5 and 24.9), 67 (25.0%) are overweight (BMI between 25 and 29.9), and 26 (9.7%) are obese (BMI ≥30) (Table 1).

**Table 1:** Socio-demographic characteristics (n = 275).

Age (year)	Under 20	33 (12%)	GPA	<2.0	35 (12.7%)
	20–25	228 (82.9%)		2.0–2.5	165 (60.0%)
	Above 25	14 (5.1%)		2.5–3.0	57 (20.7%)
Gender	Male	173 (62.9%)		>3.0	18 (6.5%)
	Female	102 (37.1%)	Current residence	Urban	251 (91.3%)
Social Status	Single	242 (88.0%)		Rural	24 (8.7%)
	In a relationship	26 (9.5%)	University Year	1	29 (10.5%)
	Married	7 (2.5%)		2	9 (3.3%)
Alcohol Use	Yes	28 (10.2%)		3	36 (13.1%)
	No	247 (89.8%)		4	49 (17.8%)
Smoking	Yes (Cigarette)	96 (34.9%)		5th	87 (31.6%)
	No	179 (34.9%)		6th	65 (23.6%)
Do you exercise at least 6.5 h per week?	Yes	144 (52.4%)	BMI (kg/m^2^)	<18.5	11 (4.1%)
	No	131 (47.6%)		18.5–24.9	164 (61.2%)
Do you know someone with diabetes?	Yes	250 (90.9%)		25–29.9	67 (25.0%)
	No	25 (9.1%)		≥30	26 (9.7%)

### 3.2 General information and risk factors regarding DM type 1 and 2

Date revealed a lack of knowledge regarding the cause of DM, only 181 (65.8%) knew that type 1 diabetes is insulin-dependent, and only 180 (65.5%) knew that type 2 diabetes is insulin resistant. We found no significant difference between pre-clinical and clinical students regarding general knowledge about DM type 1 and type 2 (Table 2).

**Table 2:** General information and risk factors about DM type 1 and 2 (n = 275).

General Information						
	Pre-clinical (n = 74)		Clinical (n = 201)		overall	p-value
	Yes	No	Yes	No	Correct answer	
Is type 1 DM Insulin-dependent?	44 (59.5%)	30 (40.5%)	137 (68.2%)	64 (31.8%)	181 (65.8%)	0.177
Is type 2 DM Insulin resistant?	50 (67.6%)	24 (32.4%)	130 (64.7%)	71 (35.3%)	180 (65.5%)	1.000
Only Type 1 DM presents with ketonuria?	48 (64.9%)	26 (35.1%)	87 (43.3%)	114 (56.7%)	135 (49.1%)	1.000
Are autoantibodies present in type 1 DM?	39 (52.7%)	35 (47.3%)	144 (71.6%)	57 (28.4%)	183 (66.5%)	0.005∗

Risk Factors								
	Pre-clinical (n = 74)			Clinical (n = 201)			overall	p-value
	Yes	No	Do not know	Yes	No	Do not know	Correct answer	
age >45 years	55 (74.3%)	7 (9.5%)	12 (16.2%)	166 (82.6%)	16 (8.0%)	19 (9.5%)	221 (80.4%)	0.126
Obesity (BMI>30 kg/m^2^)	54 (73.0%)	3 (4.1%)	17 (23.0%)	187 (93.0%)	4 (2.0%)	10 (5.0%)	24 (87.6%)	<0.001∗
Positive family history	62 (83.8%)	3 (4.1%)	9 (12.2%)	196 (97.5%)	4 (2.0%)	1 (0.5%)	258 (93.8%)	<0.001∗
History of gestational diabetes	48 (64.9%)	4 (5.4%)	22 (29.7%)	153 (76.1%)	22 (10.9%)	26 (12.9%)	201 (73.1%)	0.062
Hypertension	27 (36.5%)	18 (24.3%)	29 (39.2%)	98 (48.8%)	52 (25.9%)	51 (25.4%)	125 (45.5%)	0.069
Carbohydrate-rich diet	49 (66.2%)	8 (10.8%)	17 (23.0%)	143 (71.1%)	29 (14.4%)	29 (14.4%)	192 (69.8%)	0.430
Lack of physical activity	57 (77.0%)	5 (6.8%)	12 (16.2%)	159 (79.1%)	22 (10.9%)	20 (10.0%)	216 (78.5%)	0.709

The majority of clinical and preclinical students correctly identified DM risk factors: obesity, 187 (93%) and 54 (73%); family history, 196 (97.5%) and 62 (83.3%); lack of physical activity, 159 (79.1%) and 57 (77.0%) respectively. However, a minority of clinical and preclinical students identified hypertension, 125 (45.5%) and 27 (36.5%) respectively (Table 2).

### 3.3 DM common clinical features

**Figure 1:**
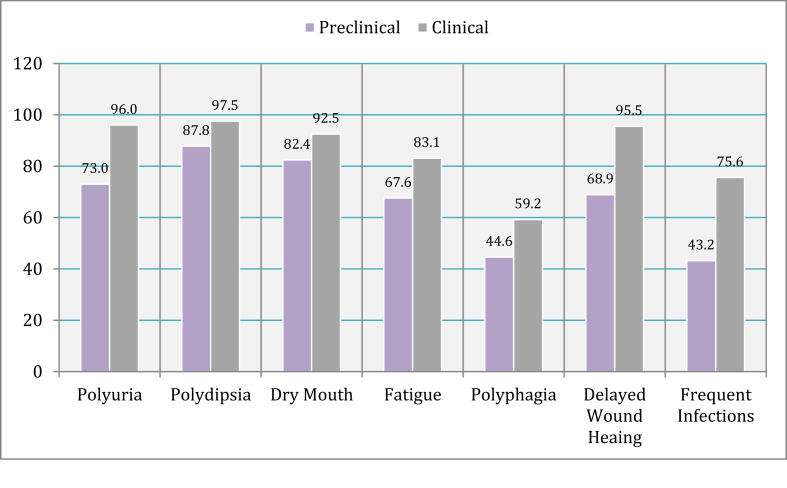
DM common signs and symptoms among preclinical and clinical medical students.

Students showed good knowledge with regards to common clinical features students, polydipsia 261 (94.9%), mouth dryness 247 (89.8%), polyuria 247 (89.8%), delayed wound healing 243 (88.4%), fatigue 217 (78.9%), and frequent infections 184 (66.9%). However, polyphagia was only reported by 152 (55.3%) students. Clinical students had a significantly higher level of awareness in comparison with preclinical students regarding polyuria (p < 0.001), polydipsia (p < 0.001)., mouth dryness (p = 0.014), fatigue (p = 0.005), polyphagia (p < 0.001), delayed wound healing (p < 0.001), and frequent infections (p < 0.001) (Figure 1).

### 3.4 DM diagnosis

With regards to diagnosing DM, both clinical and preclinical students showed a significant lack of knowledge. The diagnostic criteria of DM can include: a fasting plasma glucose test of 126 mg/dl≤, 86 (42.8%) and 18 (24.3%); oral glucose tolerance test after 2 h of 200 mg/dl≤, 74 (36.8) and 20 (27.0); HbA1c of 6.4%≤, 71 (35.3%) and 30 (40.5%) respectively (Table 3).

**Table 3:** Diagnosis.

	Pre-clinical (n = 74)	Clinical (n = 201)	Total Correct answer	T -test	p-value
Fasting plasma glucose (FGP)					
70–100 mg/dl	34 (45.9%)	74 (36.8%)	108 (39.3%)	-3.017	0.003∗
100–125 mg/dl	18 (24.3%)	28 (13.9%)	46 (16.7%)		
**126≤** mg/dl	18 (24.3%)	86 (42.8%)	104 (37.8%)		
140 ≤ mg/dl	4 (5.4%)	13 (6.5%)	17 (6.2%)		
Oral glucose challenge after 2 hours/random plasma glucose					
≤140 mg/dl	30 (40.5%)	76 (37.8%)	106 (38.5%)	-1.575	0.118
140–199 mg/dl	22 (29.7%)	44 (21.9%)	66 (24.0)		
**200≤** mg/dl	20 (27.0%)	74 (36.8%)	94 (34.2%)		
Above 400 mg/dl	2 (2.7%)	7 (3.5%)	9 (3.3%)		
Haemoglobin A1c (HbA1c):					
≤5.7%	10 (13.5%)	40 (19.9%)	50 (18.2%)	0.783	0.435
5.7–6.4 %	28 (37.8%)	69 (34.3%)	97 (35.3%)		
**≤ 6.4%**	30 (40.5%)	71 (35.3%)	101 (36.7%)		
Above 10 %	6 (8.1%)	21 (10.4%)	27 (9.8%)		

### 3.5 DM complications

Students showed a good level of awareness with regards to DM complications. Retinopathy, limb gangrene, renal failure, hyperglycaemia, peripheral neuropathy, and ketoacidosis were identified by 254(92.4%), 255(92.7%), 248(90.2%), 238(86.5%), 233(84.7%), and 229 (83.3%) students respectively. Only 159 (57.8%) and 167 (60.7%) identified cataract and hypoglycaemia as a complication of DM (Table 4).

**Table 4:** DM complications.

	Pre-clinical (n = 74)			Clinical (n = 201)			over all	chi-square	p-value
	Yes (%)	No (%)	Do not know (%)	Yes (%)	No (%)	Do not know (%)	Correct answer (%)		
Retinopathy	59 (79.7%)	5 (6.8%)	10 (13.5%)	195 (97.0%)	1 (0.5%)	5 (2.5%)	254 (92.4%)	23.516	0.001∗
Cataract	23 (31.1%)	13 (17.6%)	38 (51.4%)	136 (67.7%)	26 (12.9%)	39 (19.4%)	159 (57.8%)	33.053	<0.001∗
Renal failure	60 (81.1%)	6 (8.1%)	8 (10.8%)	188 (93.5%)	4 (2.0%)	9 (4.5%)	248 (90.2%)	10.006	0.007∗
Peripheral neuropathy	45 (60.8%)	7 (9.5%)	22 (29.7%)	188 (93.5%)	5 (2.5%)	8 (4.0%)	233 (84.7%)	45.733	<0.001∗
Ketoacidosis	48 (64.9%)	6 (8.1%)	20 (27.0%)	181 (90.0%)	4 (2.0%)	16 (8.0%)	229 (83.3%)	24.707	<0.001∗
Hypoglycaemia	38 (51.4%)	29 (39.2%)	7 (9.5%)	129 (64.2%)	45 (22.4%)	27 (13.4%)	167 (60.7%	7.83	0.019∗
Hyperglycaemia	66 (89.2%)	3 (4.1%)	5 (6.8%)	172 (85.6%)	9 (4.5%)	20 (10.0%)	238 (86.5%)	0.710	0.700
Limb gangrene	65 (87.8%)	1 (1.4%)	8 (10.8%)	190 (94.5%)	1 (0.5%)	10 (5.0%)	255 (92.7%)	3.617	0.163

### 3.6 DM prevention

**Figure 2:**
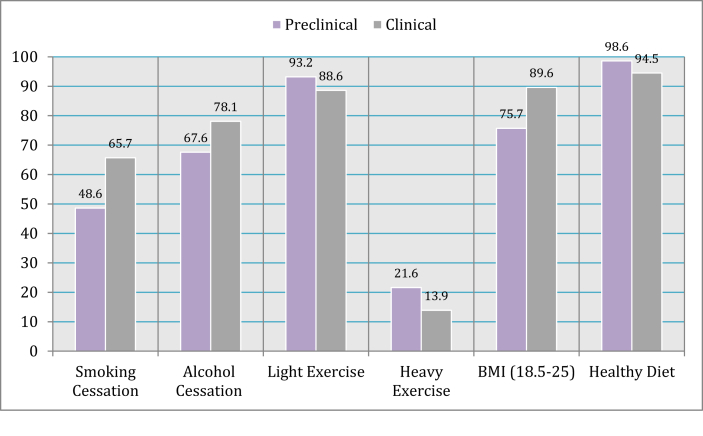
DM prevention factors among preclinical and clinical medical students.

A good level of awareness was shown with regards to DM main prevention factors, healthy diet 263 (95.6%), light exercise 247 (89.8%), and BMI within the reference range (18.5–24.9) 236 (85.8%). However, only 141 (51.3%) and 168 (61.1%) identified heavy exercise and quitting smoking as primary prevention against DM (Figure 2).

### 3.7 DM treatment and diet

Clinical students had a significantly higher level of awareness in comparison with preclinical students regarding DM treatment and recommended diet. With regards to insulin administration, clinical students 148 (73.6%) showed a higher level of awareness compared with preclinical students 38 (51.4%) (p = 0.001). A healthy diet for diabetic patients includes 35% carbohydrate +50% fats +15% protein, only 45 (16.4%) identified this diet (p = 0.003) (Table 5).

**Table 5:** DM treatment and diet.

	Pre-clinical (n = 74)	Clinical (n = 201)	over all	chi-square	p-value
**Insulin is injected through:**					
Intravenous	22 (29.7%)	42 (20.9%)	64 (23.3%)	16.540	<0.001∗
Intramuscular	14 (18.9%)	11 (5.5%)	25 (9.1%)		
**Subcutaneous**	38 (51.4%)	148 (73.6%)	186 (67.6%)		
**Diabetic diet should include:**					
50% carbohydrate +35% fats + 15% proteins	18 (24.3%)	27 (13.4%)	45 (16.4%)	9.818	0.007∗
**35% carbohydrate + 50% fats +15% proteins**	17 (23.0%)	28 (13.9%)	45 (16.4%)		
15% carbohydrate +35% fats + 50% protein	39 (52.7%)	146 (72.6%)	185 (67.3%)		

## 4 Discussion

With the increasing burden of DM and projected prevalence, it is crucial to train our future doctors in understanding and effectively managing DM. This is the first study to measure DM awareness and knowledge among medical students during the ongoing conflict in Syria. Of the students enrolled in the study, 67 (25.0%) are overweight, 26 (9.7%) are obese, and only 144 (52.4%) exercise regularly, making inactivity a serious concern among our students. This is lower compared with a Chinese study where 59.7% are physically active, 55.1% are in the normal BMI range, and only 2.9% are overweight [11]. The high percentage of overweight and obese students can be attributed to the new introduction of “Western food”, shifting from a healthy Mediterranean cousin towards an outstripped domestic production of unhealthy fried food, accompanied by our demanding growing population [12]. People who are overweight and/or lack physical activity, compared to those with BMI within the reference range, are at higher risk for many serious conditions including type 2 DM, making it a global health concern [13]. New obesity strategies should be unveiled to urge students to lose weight and increase physical activity, not only to decrease the demand on our healthcare system, but to play the part in getting our nation fit and healthy, and reduce the risks of obesity-related diseases.

We found a lack of knowledge regarding DM general information in our study. DM is either caused by insulin deficiency or a combination of both insulin deficiency and resistance, awareness of these mechanisms is key to understanding the diagnosis and management of patients [1]. 181 (65.8%) and 180 (65.5%) know that type 1 diabetes is Insulin-dependent, and type 2 is a combination of Insulin resistance and Insulin-dependent, respectively. This was higher in comparison with a study in Jordan [14]. The majority of students failed to identify ketonuria as a manifestation of type 2 DM, among insulin-dependent patients. Clinical students have completed the theory aspect of physiology, pathology, and pharmacology regarding DM and will be exposed to the clinical settings of diabetic patients presenting with both acute and chronic complications. This lack of knowledge behind the mechanism of DKA will place patients at risk for complications and requires further addressing on our behalf.

Our students have good knowledge regarding risk factors, the majority identified old age, obesity, and a family history of diabetes as risk factors for DM. Our results were higher in comparison with Islamabad [8] and America [15]. A lack of knowledge was identified regarding a carbohydrate-rich diet and sedentary lifestyle as risk factors for DM, which is less than a study conducted in Southern Sri Lanka [16]. Both clinical and preclinical students had similar levels of awareness regarding the majority of risk factors. DM risk factors are known to everyone as it is an important topic in every healthcare, community, and social area, which can be the reason behind the knowledge among our students [17].

Early recognition of DM clinical features plays a role in preventing serious complications of the disease [18]. Polyuria, polydipsia, and fatigue are the main classical symptoms of DM [18]. Our study showed higher levels of awareness in comparison with studies conducted in Jordan [14], Saudi Arabia [19], Pakistan [20], and United Arab Emirates (UAE) [21]. Delayed Wound healing can sometimes be overlooked by both healthcare workers, and patients, which can be problematic if unidentified and therefore untreated [22]. Both clinical and preclinical students showed higher awareness regarding delayed wound healing as a clinical feature of DM in comparison with a study in Pakistan [20], and UAE [21]. Patients with DM are at higher risk of frequent infections because of their deficient immune systems [23]. Our students showed higher awareness regarding this matter in comparison with a study in UAE [21]. Clinical students showed a significantly higher level of awareness in comparison with preclinical students; this is attributed to the clinical experience in hospitals, and that the endocrinology curriculum is taught in the fifth year of medical school.

Our study revealed a significant lack of knowledge regarding diagnostic criteria among both clinical and preclinical students. DM diagnostic criteria for fasting plasma glucose (FGP), oral glucose challenge after 2 h, and HbA1c, was higher in our study in comparison with a study in Pakistan [8], but lower than a study in South India [24]. These results raise an alarming flag since a big portion of our students lack the knowledge of the diagnostic tests for diabetes. A well-timed accurate diagnosis and early intervention are critical to slow down and mitigate complications of DM. The onset of type 2 DM happens 4–7 years before clinical diagnosis where clinical inertia by physicians providing health care is the reason behind delayed diagnosis [25, 26].

The execution of DM prevention in communities has been proven to prevent type 2 DM [27]; modifiable lifestyle changes are required to achieve this goal [28]. Smoking has a dreadful effect on DM complications, especially microvascular disease [29]. 96 (34.9%) of students smoke in our study, and 168 (61.1%) were aware that smoking cessation is a strategy of DM prevention, which was similar to other studies in India [30], and in UAE [21]. Physical activity has Physiological benefits including prevention and management of diabetes [31]. Our study revealed high levels of awareness regarding physical activity as a preventative measure against type 2DM, which was similar in comparison with a study in Pakistan [20]. However, even knowledgeable participants, this knowledge is not translating to the practical application of evidence-based recommendations about exercise protocols, due to the lack of physical activity among our students.

DM complications are responsible for significant morbidity and mortality [32]. Ocular microvascular complications include glaucoma, cataracts, and retinopathy [33]. DM Retinopathy is the leading cause of blindness in middle-aged and elderly people [34]. Our students showed a higher level of awareness in comparison with studies in India and Ghana [30, 35], but was similar to a study in Saudi-Arabia [9]. 40% of DM patients develop diabetic nephropathy, inevitably leading to chronic renal failure and thus requiring dialysis treatment [36]. Students demonstrated a higher level of awareness in comparison with India [30], but lower than Saudi-Arabia [9]. The progression of DM peripheral neuropathy is inversely proportional to life expectancy, hugely impacting the quality of life [37]. The majority of students correctly identified peripheral neuropathy as a DM complication, showing higher levels of knowledge in comparison with studies in Saudi-Arabia and Jordan [14, 19]. These findings attract attention to the impact of DM knowledge regarding the progression of debilitating conditions, hopefully leading to increased recognition of complications and early intervention.

DM treatment depends on the type and is individualized according to each patient [38]. Only 186 (67.6%) students knew that insulin is given via subcutaneous injections, which signifies a need for interventions to address the deficiency in knowledge among our students. This will be required at multiple levels in our undergraduate curriculum and continuous medical education for physicians to reinforce the practical consequences of the prediabetes recommendations and DM management guidelines.

## 5 Limitations

There are several limitations common in the study design and sampling method. Due to the nature of the study type, self-reported findings, particularly in relation to variables such as BMI and exercise where respondents may be tempted to provide more socially desirable answers, can affect the outcomes of the study. Another limitation is the lack of representation of the entire population affecting external validity. Estimating sampling variability and identifying possible bias was another limitation. Also, random plasma glucose was not measured to determine the glucose profile of our students. To get a more inclusive picture, questions about the medications used in the management of DM should have been included. Only a few papers were published to assess medical students' knowledge in the medical literature, especially in the MENA region, making it hard to compare our students' knowledge with others**.**

## 6 Conclusion

This study demonstrates that medical students at the Syrian Private University have good knowledge around DM with the highest level of knowledge being among clinical students and lowest among pre-clinical students. The data revealed a lack of knowledge with regards to general information and diagnostic criteria for DM. Educating medical students on screening, diagnostic criteria, and treatment guidelines for DM can bring us one step closer to managing the DM epidemic in Syria. This can be achieved through revaluation of teaching methods, updating curriculum, and input from the Syrian Ministry of Education.

## Declarations

### Author contribution statement

Fatema Mohsen, Homam Safieh and Humam Armashi: Performed the experiments; Wrote the paper.

Mosa Shibani: Performed the experiments; Analyzed and interpreted the data.

Hlma Ismail and Mhd Amin Alzabibi: Performed the experiments.

Bisher Sawaf: Conceived and designed the experiments.

### Funding statement

This research did not receive any specific grant from funding agencies in the public, commercial, or not-for-profit sectors.

### Data availability statement

Data included in article/supplementary material/referenced in article.

### Declaration of interests statement

The authors declare no conflict of interest.

### Additional information

No additional information is available for this paper.

## Figures and Tables

**Table:** 

Socio-demographic Characteristics	
**Age (year):**⬜ Under 20 ⬜ 20–25 ⬜ Above 25	**Mother's Education:** ⬜ Primary ⬜ Secondary ⬜ High School ⬜ University ⬜ Graduate
**Gender:** ⬜ Male ⬜ Female	**Current residence:** ⬜ Urban ⬜ Rural
**Social Status:** ⬜ Single ⬜ In a relationship ⬜ Married	**Smoking:** ⬜ Yes ⬜ No
**College Year:** ⬜ 1st ⬜ 2nd ⬜ 3rd ⬜ 4th ⬜ 5th ⬜ 6th	**Alcohol Use:** ⬜ Yes ⬜ No
**GPA:** ⬜ < 2.0 ⬜ 2.0–2.5 ⬜ 2.5–3.0 ⬜ > 3.0	**Do you exercise at least 6.5 h per week?** ⬜ Yes ⬜ No
**BMI (kg/m**^**2**^**):** ⬜ < 18.5 ⬜ 18.5–24.9 ⬜ 25–29.9 ⬜ ≥ 30	**Do you know someone with diabetes?** ⬜ Yes ⬜ No

**Table:** 

Knowledge about diabetes mellitus:			
	Yes	No	DNK
**General knowledge**			
Is type 1 DM Insulin-dependent?			
Is type 2 DM Insulin resistant?			
Only Type 1 DM presents with ketonuria?			
Are autoantibodies present in type 1 DM?			

**Risk Factors**			
age >45 years			
Obesity (BMI>30 kg/m^2^)			
Positive family history			
History of gestational diabetes			
Hypertension			
Carbohydrate-rich diet			
Lack of physical activity			

**Signs and symptoms**			
Polyuria			
Thirst (polydipsia)			
Dry mouth			
Tiredness, fatigue, lethargy			
Persistent hunger (Polyphagia)			
Delayed wound healing			
Frequent infections			

**Complications**			
Retinopathy			
Cataract			
Renal failure			
Peripheral neuropathy			
Ketoacidosis			
Hypoglycemia			
Hyperglycaemia			
Limb gangrene			

Treatment and Diet
**Insulin is injected through:**⬜ Intravenous ⬜ Intramuscular ⬜ Subcutaneous
**A diabetic diet should include:**⬜ 50% carbohydrate +35% fat +15% protein⬜ 35% carbohydrate +50% fat +15% protein⬜ 15% carbohydrate +35% fat +50% protein
